# Groups as fictional agents

**DOI:** 10.1080/0020174X.2023.2213743

**Published:** 2023-05-18

**Authors:** Lars J. K. Moen

**Affiliations:** Department of Philosophy, University of Vienna, Vienna, Austria

**Keywords:** Group agency, individual agency, judgment aggregation, ontological parsimony, strategic behaviour

## Abstract

Can groups really be agents or is group agency just a fiction? Christian List and Philip Pettit argue influentially for group-agent realism by showing how certain groups form and act on attitudes in ways they take to be unexplainable at the level of the individual agents constituting them. Group agency is therefore considered not a fiction or a metaphor but a reality we must account for in explanations of certain social phenomena. In this paper, I challenge this defence of group-agent realism by showing how it is undermined by individual-level analysis of how individuals interact within groups. While group agency can be a useful fiction, real agents are at the individual level, not the collective level.

## 1. Introduction

Groups of individuals are often talked about as agents with beliefs and desires. Corporations, political parties and other groups have goals they want to achieve and act on their beliefs about how to do so. But while we might agree that this view of groups as agents can be useful for various reasons, a more controversial matter is whether a group can *really* be an agent in the way an individual is. For ‘eliminativists’ about group agency, group behaviour is explainable in terms of individual agency, and group agency can be no more than a useful fiction, or metaphor, referring to individuals’ collective behaviour. Group-agent realists, on the other hand, argue that failing to appreciate certain groups as irreducible agents can impair our understanding of their behaviour and place in the social world.

In this paper, I consider whether certain groups really are agents, or whether group agency is a mere fiction. I do so based on a principle of parsimony saying we should attribute no more intelligence, rationality, or consciousness to a system than is necessary for understanding its behaviour (Dennett 1976, 182). If we can make sense of how a group behaves without attributing mental states to it as an agent with its mind, but rather to individuals at a more basic level, group-agent realists will have no strong case against eliminativist explanations of group behaviour. So, the question of the ontological status of group agency is to be settled on whether an explanation of group behaviour is reducible to the level of individual agency.

Among the accounts of group-agent realism, Christian List and Philip Pettit’s defence of this position is particularly interesting because it is meant to respect this principle of parsimony. As we shall see, they argue that certain groups are inevitably capable of autonomously forming their own mental states. To understand their behaviour, we must therefore treat them as agents with their own minds (List and Pettit 2011, 8). List and Pettit are thus committed to ascribing no more mentality to groups than is needed to explain group behaviour. And they criticise other accounts of collective agency in the philosophical literature for ‘over-ascription of agency’ (List and Pettit 2011, 215–216n18).^1^ Individuals, on List and Pettit’s view, do not constitute a collective agent merely by acting together on intentions they form based on their expectations of how others will behave towards them.

To briefly explain why List and Pettit see a need for group agency, suppose a group is to decide on a set of logically interconnected propositions, where the judgment, or judgments, of one or more propositions entails a judgment of another proposition. The judgments of *p* and if *p* then *q* (*p* → *q*), for example, entail a judgment of *q*. Each group member holds consistent judgments of these propositions so that no attitude precludes another. But when we add up the members’ judgments, we might nonetheless find the aggregated judgments to be inconsistent. The group therefore cannot adopt its members’ judgments. It must instead form a set of judgments few, or perhaps none, of the group members hold. These judgments cannot be attributed to these individuals; they must be attributed to the group itself. The group is therefore understood to necessarily possess considerable control over its own judgment formation, and we shall fail to account for this important fact, the argument goes, unless we see the group as an agent in its own right. We cannot understand groups’ behaviour as strictly based on individuals’ attitudes.

Group agency is therefore said to be non-redundant in explanations of social phenomena. List and Pettit thus try to demonstrate the reality of group agency without introducing more collective-level mentality than is necessary for explaining how groups operate in the social world. Their commitment to parsimony makes their defence of group-agent realism a particularly strong and interesting challenge to eliminativists rejecting the reality of group agency.

As I take on this challenge, I accept the possibility of inconsistent aggregates of individuals’ judgments, and that a group therefore may have to form judgments not strictly reflective of its members’ judgments. I will deny, however, the alleged implication that the group must therefore control its own judgment formation. I argue that collective judgment formation is controlled by individuals, even when the aggregates of group members’ judgments are inconsistent. I do so by demonstrating the significance of individuals’ agenda-setting power and capacity for strategic expression of judgments. That is, individuals controlling what propositions appear on the agenda can effectively determine the outcome. And individuals can misrepresent their judgments to help bring about the best possible outcome for themselves. Since, as we shall see, such manipulation is virtually always possible and cannot be assumed away, we should always account for individuals’ motivations for bringing about a collective outcome.

The need for such analysis of individuals’ behaviour within the group structure leads to the conclusion that List and Pettit’s commitment to ontological parsimony is incompatible with group agency, and their defence of group-agent realism therefore fails. But for various reasons, it may still be useful to talk about groups as if they were agents. Pettit (2014) identifies three such reasons. We can use group agency as a figurative way of speech, thereby considering it an ‘expressive fiction’. And it can be a ‘pragmatic fiction’ providing a convenient way of articulating the significance and legitimacy of collective action. It can also be a ‘theoretical fiction’ useful for explaining and predicting individuals’ collective behaviour. I shall argue that such usage is grounded on individual-level explanations of group behaviour, and that group agency therefore cannot be more than a useful fiction.

I explain these uses of group agency as a fiction in Section 2, before I turn to List and Pettit’s autonomy argument for realism in Section 3. In Section 4, I show how this argument fails to appreciate that group members are themselves strategically behaving agents. This leads me to defend fictionalism in Section 5. But as I show in Section 6, individuals’ strategic behaviour can undermine their group’s agential ability to form true beliefs, which not only undermines group-agent realism but also limits the usefulness of group agency as a theoretical fiction.

## 2. Fictional group agency

I shall follow List and Pettit (2011, 20) in understanding an agent as a system or entity with representational states, or beliefs, about what its environment is like; motivational states, or goals or desires, about how it would like things to be; and a capacity to intervene in its environment so as to achieve its goals or to satisfy its desires. We treat a group as an agent to the extent that it makes sense to say it has desires it acts to satisfy based on its beliefs about the environment in which it operates.

We may talk about certain groups in this manner while holding that group agency is merely a useful fiction. In this section, I briefly look at the three ways of treating group agency as a useful fiction that Pettit (2014) identifies. On these views, we ascribe beliefs and desires to groups, but these attitudes are taken to *really* just reflect group members’ attitudes. Talk of group agency, therefore, does not refer to an actual agent, but rather to a collection of individual agents. Pettit (2014, 1643–1644) accepts that such talk is sometimes rightfully considered metaphorical, as in the cases of the bond market and the electorate. But we shall see that he considers certain groups to be organised and to function in such a way as to qualify as real agents.

As an expressive fiction, group agency is a metaphor useful for describing group behaviour. Attitudes expressed by the group’s spokesperson are ascribed to the group, but they are not really the group’s self-represented attitudes. They are instead mere representations of the group members’ attitudes. John Austin (1869, 364) takes this view when he refers to group agency just ‘for the sake of brevity of discussion’. And Anthony Quinton (1976, 17) understands talk of group agents as ‘plainly metaphorical’. Ascribing mental states to a group is, in Quinton’s view, really just an indirect way of ascribing them to its members. So, when we say that a corporation did something or a political party holds a particular view, we are actually just referring to individuals within these groups.

As a pragmatic fiction, group agency refers to one or more individuals representing the group. This view no doubt holds in the case of a dictator, as the group’s attitudes are then obviously reducible to those of the dictator. When the group acts, it acts on the will of this individual. In such cases, we clearly refer to the dictator’s attitudes and decisions when we say that the group holds this and that attitude and makes this and that decision. But on the pragmatic-fiction view, a reductionist account of group attitudes is believed to be available also when there are several individuals involved in the decision making.

Pettit (2008, Ch. 5; 2014, 1647–1649) attributes this view to Thomas Hobbes. For Hobbes (2008, Ch. 16), certain groups are agents ‘by fiction’ as they can speak only through a spokesperson. A group does not have its own mind or voice; it is only a representation of the will of a dictator or a committee or some other collective decision-making body. In the latter case, Hobbes (2008, 109) takes a majoritarian view by noting that ‘the voice of the greater number, must be considered as the voice of them all’. Since Hobbes, this has been an influential view of the state in Western political thought (Pettit 2008, 82; Skinner 1989). Attributing agency to such a group is believed to enhance its significance and legitimacy. But the group, on this view, is not really an agent; it does not itself possess a mind or a voice of its own. It is more accurately understood to function on the will of an authorised individual or subgroup.

Finally, as a theoretical fiction, groups are modelled as agents for the sake of explaining or predicting their behaviour. We take a group to hold desires it wants to satisfy and to act on beliefs about its environment in the pursuit of such satisfaction. International-relations theorists, for example, commonly model states as interacting agents (Kydd 2015, ch. 2). Country A’s ‘desire’ to reduce its defence spending, let us say, may depend on its ‘beliefs’ about whether Country B will cut its defence spending. But these theorists usually do not take a country or its government to actually be an agent. Their ascription of agency to collective entities is instead meant as a helpful way of interpreting their behaviour. The collective agency is no more than an abstraction from the attitudes of individuals who *really* determine how the group functions. Group agency is therefore dispensable in a fine-grained individualistic theory, and only individuals are considered real agents non-redundant in explanations of social phenomena.

## 3. The irreducibility argument for realism

Pettit (2014) rejects each of these fictionalist views. By scrutinising what we refer to when we give agential states to groups, Pettit and other group-agent realists deny that these states can be ascribed to individuals. Some groups, they argue, can form and act on their own irreducible mental states. And since we then cannot explain their behaviour by ascribing mental states to lower-level entities, we are right to consider them real agents.

To understand the argument for group-agent realism, let us first note that it concerns groups forming attitudes that might differ from those of their members. The outcome of a discussion between group members, for example, could be attitudes the members accept as their group’s attitudes although they do not actually hold these attitudes themselves.^2^ This might be an attractive way of ensuring the group makes reasonable decisions (Moen forthcoming). And Pettit (2007, 512; 2009, 81–88; 2014, 1650–1652; 2018, 20–21) considers such group deliberation a plausible basis for group agency.

Pettit here especially focuses on cases where a group cannot always adopt the judgments held by most of its members because majority judgments might conflict. Suppose we have an agenda with interconnected propositions, such as *p*, *p* → *q*, and *q*. We assume that each individual holds complete and consistent judgments, so that she or he judges on each proposition and none of these judgments precludes any of the individual’s other judgments. Nonetheless, the aggregates of all the individuals’ judgments will sometimes be inconsistent (Table 1). Pettit (2008, 82–83; 2014, 1650) is therefore right when he says Hobbes and others are mistaken to believe groups can have a strictly majoritarian voice. No procedure can ensure group-level rationality and responsiveness to the members’ attitudes. We can only have one or the other, not both. Pettit (2001, 272) calls this ‘the discursive dilemma’.^3^

**Table 1.** Each group member submits complete and consistent attitudes towards three interconnected propositions. The majority attitudes are nonetheless inconsistent.

	*p*	*p* → *q*	*q*
Individual A	True	True	True
Individual B	True	False	False
Individual C	False	True	False
**Majority**	**True**	**True**	**False**

To deal with this problem, the group members can form judgments in a process of deliberation, where they discuss how to deviate from a majority judgment to ensure collective rationality. List and Pettit (2011, 61–64) view individuals engaging in a process of ensuring a consistent set of group attitudes as parts of a group mind that reflects on its own attitudes to sort out any inconsistency (see also Tollefsen 2002a, 401).

There is, of course, a sense in which the group members here come together to form a set of attitudes that differs from the aggregates of their attitudes. But while the set of judgments formed in the deliberation may reflect no individual’s judgment set, its formation is nonetheless controlled by individuals. Based on the information the group members gather about each other’s attitudes, they can find the outcome most in line with their preferences. We can therefore explain the outcome in terms of individuals’ beliefs and desires. No one individual may be in control, but the reasoning leading to the collective outcome occurs in individuals' minds. There is no need, then, to introduce a group mind.

The argument for group-agent realism looks stronger with a procedure that rigorously applies what List (2004) calls a functionally explicit priority rule. Such a procedure aggregates individuals’ attitudes and mechanically produces a consistent outcome by giving priority to a certain proposition, or propositions. One such procedure is the premise-based procedure, which ascribes the majority judgments of the premises – so, in the example, *p* and *p* → *q* – to the group, and then the judgment of the conclusion that follows from these judgments – in the example, *q*. With the profile of individuals’ judgments in the example, we then get the judgment set {*p*, *p* → *q*, *q*}.

Alternatively, we can employ the conclusion-based procedure by taking votes directly on the conclusion. To ensure judgments of all propositions, this procedure depends on a supplementary mechanism since more than one set of judgments of the premises may be consistent with the prioritised judgment of the conclusion. In the example, this procedure results in a rejection of *q* (¬*q*) and support for no more than one of *p* and *p* → *q* – that is, {¬(*p* ∧ *p* → *q*), ¬*q*}.

Other procedures can also deal with the inconsistency problem, but I will restrict my focus here to the premise-based and conclusion-based procedures. This will suffice for the discussion below, which can be extended to other procedures. We can evaluate the strengths and weaknesses of these different procedures. The premise-based procedure, for example, might be superior for reaching decisions for the right reasons – that is, sound and supportive reasons (Bovens and Rabinowicz 2006; Pettit and Rabinowicz 2001). The conclusion-based procedure, on the other hand, is more efficient. The point here, however, is that the two procedures can deliver different outcomes based on the same profile of individual judgments (Table 2).

**Table 2.** Two procedures generate different sets of group attitudes based on the same profile of individual attitudes.

Premise-based procedure	*p*, *p* → *q*, *q*
Outcome-based procedure	¬(*p* ∧ *p* → *q)*, ¬*q*

So, collective judgments cannot simply depend on individuals’ judgments, they also depend on which procedure is employed. This observation leads List and Pettit to see a reflective group mind. The procedure does not just produce an outcome based on the individuals’ judgments; it must ‘reflect’ on these judgments to ensure a consistent set of collective judgments. If the aggregates of individuals’ judgments are consistent, then they will be the group’s judgments. But if they are inconsistent, the reflective group mind will form a different set of judgments. For List and Pettit, this means the group is inevitably in control of its judgment formation. They understand it to function on its desire to make its beliefs consistent. The group, they say, exercises a ‘surprising degree of autonomy’ (List and Pettit 2011, 59).

This autonomy argument is crucial for demonstrating the non-redundancy of group agency, and therefore for showing how ontological parsimony is compatible with group-agent realism. We cannot explain how the group came to act on attitudes formed by these procedures by looking at the group members’ attitudes. By ignoring the significance of the ‘group mind’, the argument goes, we shall fail to explain the group’s behaviour.

## 4. Individual control

A fictionalist view of group agency is untenable insofar as it entails that group attitudes are invariably straightforward aggregates of individual attitudes. The general result that no procedure that fairly represents individuals’ attitudes can ensure collective-level consistency settles that matter. Crucially, however, this does not mean we must invoke collective mentality to explain collective decision making. The aggregation problem, in other words, does not by itself falsify the fictionalist position on group agency and vindicate the realist one. To reach that conclusion, one must also show that individuals do not control the collective outcome; that the group is to a significant degree autonomous, as List and Pettit claim. In this section, however, I show why group members remain in control despite the impossibility of a procedure giving fair consideration to their attitudes.

As noted above, the challenge for group-agent realists is to show that we must ascribe mental states to groups to explain their behaviour. As long as we can explain group behaviour in terms of individual agency, we are not warranted to treat group agency as more than a fiction. We have seen how collective-level mechanisms can produce different outcomes based on the same profile of individuals’ attitudes, and that List and Pettit take this to demonstrate the reality and significance of a reflective group mind. But I shall formulate an individual-level explanation the success of which shows how aggregation problems do not make group agency necessary in social explanations.

I do so by taking the perspective of an individual member of the sort of group to which List and Pettit ascribe agency. In accordance with the principle of parsimony, I start at the level of individual agency and see how far I get in explaining group behaviour without ascribing agency at the group level. What we notice from the individual’s point view is that within the group, the individual finds herself in a strategic situation. That is, she interacts with other individual agents, and she cannot directly determine the outcome of this interaction. The collective outcome will instead depend on how her actions combine with those of the other group members. Knowing this, the individual’s behaviour will depend on the consequences she expects of her actions given what she knows about her social environment and her place within it.

In the analysis of individuals’ behaviour within their group, it is particularly important that the collective decision procedure is manipulable. In general, a procedure is manipulable when what outcome it produces depends on how an individual sets the agenda (agenda control), or when an individual can contribute to a better outcome (from her point of view) by misrepresenting her sincere attitudes (strategic voting). An important result in social choice theory is that with three or more possible outcomes, which is virtually always the case, any non-dictatorial decision procedure is manipulable (Gibbard 1973; Satterthwaite 1975). Dietrich and List (2007) further demonstrate the ubiquity of manipulability in judgment aggregation.^4^ I will now show how this result enables us to understand collective outcomes – even in the cases of inconsistency List and Pettit rely on – in terms of individuals acting on their preferences.

Consider again the example presented in Table 1. Someone has to decide which propositions appear on the agenda, and this individual can choose propositions she expects to result in her preferred outcome. Suppose Individual B sets the agenda. B believes that *p* is true, *p* → *q* is false, and that *q* is false. Suppose B places just *q* on the agenda, thus choosing a conclusion-based procedure where only the outcome is voted on. As we saw in the previous section, the outcome is then a rejection of *q*, since B and C form a majority against *q*. We can then understand B to act on a preference for her group rejecting *q – q* is her ‘proposition of concern’. But suppose instead B places all the propositions the agenda and employes the premise-based procedure. The group then accepts all the propositions. We can explain this outcome as B preferring the group to accept her view of the premise *p* – *p* is her proposition of concern.

We can see the significance of agenda setting also in cases where the majority judgments are consistent. The consistency result can be understood as an expression of the agenda setter's power. Suppose Individual B selects the set of propositions *r*, *r* → *q*, and *q*, and that majorities reject all of these propositions. In this case, we can interpret the outcome as a result of B choosing propositions to ensure that the group rejects *q* even with the premise-based procedure.

We can now see how we can understand the outcome – whichever way it goes – as the agenda setter’s construction, and not as the output of a reflective group mind. Now, it might be that the agenda setter chooses not to exercise her power. Perhaps she considers it immoral to take an opportunity no other group member has to influence the collective decision making. She might therefore act on a principled commitment to one or another procedure. But this takes nothing away from the fact that her decisions have a crucial impact on the outcome. We miss out on this crucial insight by considering the group itself as an agent autonomously forming its judgments.

Another dimension of individual-level control becomes apparent when we consider individuals’ capacity to express attitudes strategically (Moen 2023).^5^ Now we suppose the individual is an ordinary voter without agenda-setting power. The important thing to notice here is that by voting for one outcome, the individual can indirectly contribute to a different outcome (Braham and van Hees 2011). This phenomenon is actually what List and Pettit’s argument for group-agent realism depends on. In the example, Individuals B and C’s sets of judgments contribute to the collective-level inconsistency with the group judging that *p*, *p* → *q*, but not *q*.

If they vote accordingly, and if the premise-based procedure is employed, the group will accept *q*. But even if we set aside the significance of agenda setting, we should not interpret this outcome as a product of a group mind. B and C know they could have voted differently, and they know that by voting this way with a premise-based procedure, they can indirectly contribute to a collective judgment of *q* they do not hold. We should then understand their voting behaviour to be based on a preference for their group adopting their judgments of one of the premises (*p* or *p* → *q*). Had their proposition of concern instead been *q*, they would have voted differently on one of the premises so as not to contribute – given the premise-based procedure – to their group accepting *q*.

With awareness of individuals behaving strategically in response to the decision procedure and how they expect others to behave, we can understand their group’s behaviour in terms of individuals acting on their preferences. When they care more about a premise on the agenda (in our example, *p* or *p* → *q*), they have reason-oriented preferences. When they care more about the conclusion (*q*), they have outcome-oriented preferences (Dietrich and List 2007, 289–291).

With the premise-based procedure, individuals with reason-oriented preferences will not misrepresent their judgments (Dietrich and List 2007, 290). This is because the procedure accepts the majority judgments of the premises. We can therefore understand the outcome of the premise-based procedure, where the group accepts *q* despite only A supporting *q*, to be a result of B and C acting on their reason-oriented preferences. If they had outcome-oriented preferences, they would have misrepresented their judgments of *p* or *p* → *q* so as not to contribute to the collective decision in favour of *q*. B could have misrepresented her judgment of *p* to make it impossible for her to contribute to an inconsistency and an outcome conflicting with her view of *q*. And C achieves the same by voting strategically against *p* → *q*. As we see in Table 3, the result would then be a set of majority judgments consistent with the judgment of *q* that B and C prefer.

**Table 3.** The group applies the premise-based procedure and individuals B and C vote strategically based on their outcome-oriented preferences.

	*p*	*p* → *q*	*q*
Individual A	True	True	True
Individual B	True False	False	False
Individual C	False	True False	False
**Majority**	**True False**	**True False**	**False**

With the conclusion-based procedure, individuals with outcome-oriented preferences have no incentive to vote strategically, as the group will then adopt the majority judgment of the conclusion. In the example, where all three propositions are on the agenda and the conclusion-based procedure delivers the result against *q*, which B and C supports, we can understand B and C to act sincerely due to their outcome-oriented preferences. They would have revealed reason-oriented preferences, on the other hand, had they voted strategically for *q*. That is, B’s proposition of concern would have been *p* and C’s proposition of concern *p* → *q*. To express consistent judgments, B would then also have voted for *p* → *q* and C would have voted for *p*, as we see in Table 4.

**Table 4.** The group applies the conclusion-based procedure and individuals B and C vote strategically based on their reason-oriented preferences.

	*p*	*p* → *q*	*q*
Individual A	True	True	True
Individual B	True	False True	False True
Individual C	False True	True	False True
**Majority**	**True**	**True**	**False True**

This analysis of actual and counterfactual outcomes demonstrates the significance of individuals’ preferences. We might, as we have seen, get different outcomes from the same profile of individuals’ judgments. But we can now see how individuals’ preferences can nonetheless explain these outcomes. If we treat the procedure as an autonomous group mind, we fail to see that which procedure is employed affects how individuals vote. As illustrated in Table 5, it is individuals’ preference orientations rather than the procedure that determine the outcome.^6^

**Table 5.** Group members’ preference orientation, and not the group’s decision procedure, determines the group judgments.

	Reason-oriented preferences	Outcome-oriented preferences
Premise-based procedure	*p*, *p* → *q*, *q*	¬*p*, ¬*p* → *q*, ¬*q*
Outcome-based procedure	*p*, *p* → *q*, *q*	¬*p*, ¬*p* → *q*, ¬*q*

## 5. Fictionalism defended

By viewing individuals as agents with a capacity to respond strategically to their social environment, we realise they have more control over their group’s attitude formation than is accounted for in group-agent realism. We miss this important insight if we try to explain the group’s behaviour by treating it as an irreducible agent autonomously forming its own attitudes. We should therefore instead pursue an explanation reducible to individual psychology. And according to the principle of parsimony, that means groups do not qualify as real agents.

This does not prevent us from talking about certain groups as if they were agents. It may not always be convenient or necessary to carefully explain how individuals combine to produce a decision. We can then conveniently refer to it as the group’s decision and say that it expresses the group’s belief or desire. But we are then using group agency as an expressive fiction. The beliefs and desires we ascribe to the group may not be aggregates of the group members’ sincere attitudes. But we have seen how this result can be understood as the agenda setter’s intended outcome, or as the collective beliefs and desires the group members prefer out of the possible sets of attitudes within the constraints of their social environment.

Individual-level control of collective outcomes also implies that we can use group agency as a pragmatic fiction, as the agency we ascribe to a group for the sake of legitimacy, significance, or some other reason refers, as a matter of fact, to the group members. This view might not be exactly what Hobbes had in mind when he discussed fictional group agents. According to Pettit, Hobbes and later political thinkers failed to see that majoritarian decision making can be inconsistent. This important result is laid plain in contemporary social choice theory, including in List and Pettit’s work on judgment aggregation. But we have seen how individuals nonetheless remain in control over the decision making. We may not be able to explain a group’s decision making only by looking at the judgments individuals sincerely hold, but nor should we expect that. We must take into consideration what motivations individuals might have when they contribute to an aggregation function producing collective judgments. By doing so, we can, as we have seen, explain the outcome as a result of individuals acting on their preferences. And the justification for collective action one can achieve by pragmatically treating the group as if it were an agent can indeed explain individuals’ motivations to control it. Ascription of agency to groups, therefore, refers to a fiction, and potentially a pragmatically useful one.

We can also use group agency as a theoretical fiction. On this view, we ascribe agency to a group for the sake of interpreting its behaviour. But if this strategy succeeds, Pettit (2014) argues, there is nothing fictional about our account of agency. Here Pettit relies on Daniel Dennett’s (1971, 1987, 1991) interpretational account of agency: if a system can be interpreted as an agent, it is an agent.^7^ Dennett, it should be mentioned, talks about agential, or intentional, systems in general, and he does not defend group agency specifically. But this model works for certain groups, according to List and Pettit. We can best interpret these groups’ behaviour, they argue, by ascribing beliefs to them that they rationally ought to have given the environment they are in, and we then expect them to have desires they seek to satisfy in accordance with these beliefs. This is to take what Dennett calls ‘the intentional stance’ towards groups.

Now, we can treat a group as an agent with beliefs and desires for some theoretical purpose – to model its interaction with other groups, for example – but still consider group agency no more than a useful fiction. Its actions reliably conform to a pattern of representational states across time and different contexts. This predictability enables it to make commitments and to function in the society as any other agent. For group-agent realists, such successful attribution of attitudes implies that the group is an agent, at least insofar as the formation of these attitudes is beyond individuals’ control. We have seen, however, that individuals do control their groups in ways requiring us to understand their group’s behaviour at the individual level. So, at least in accordance with the principle of parsimony, the possibility of interpreting a group from the intentional stance is insufficient for declaring group agency a reality.

As noted above, List and Pettit accept the principle of parsimony, but they deny that it excludes group agency from the domain of reality. And if their autonomy argument for group-agent realism were sound, then it would be impossible for the group to act strictly on its members’ attitudes. The group would then necessarily be able to act on a pattern elusive at the individual level. Our ability to understand its behaviour would have been significantly impaired, and List and Pettit would have been right to declare the group a real agent. Just as we cannot understand individuals’ behaviour at the level of neurons, they argue, we cannot understand a group’s behaviour by reducing it to individuals.^8^ Individuals can perform consistently in accordance with an intentional pattern despite differences in their neuronal configurations. Analogously, the group can behave reliably in this sense by employing procedures adjusting its attitudes to conform to an interpretable pattern despite variances at the individual level. Group agency would then be non-redundant in explanations of group behaviour (List and Pettit 2011, 6–10).

We have seen, however, that the autonomy argument fails, and that group agency, therefore, is redundant in social explanations. What is missed in the neuron–individual analogy is that individuals are strategic agents, while neurons are not. When we appreciate this fact, we see how individuals control their group’s behaviour despite the possibility of inconsistent majority attitudes. We can therefore interpret the group’s behaviour from an individualist perspective. It is indeed only by taking the intentional stance towards the group members that we can understand why they behave the way they do: they act to contribute to their preferred outcome and to avoid contributing to an outcome they do not prefer. It is therefore the treatment of groups as irreducible agents that impairs our ability to explain how they function.

So, we again see how the previous section’s analysis of group members’ strategic behaviour disqualifies groups from real agency. It is instead a theoretical fiction: it might be convenient to interpret it as an agent, but we understand its behaviour more accurately by recognising that it is reducible to its members.

## 6. False beliefs

The reducibility of explanations of group behaviour to the individual level shows why group agency is a fiction, albeit a potentially useful one. One reason why this observation is important is that it enables us to see limitations of the group-agency model. In this last section before I conclude, I demonstrate one way in which group agency, even as a fiction, is limited in a way individual agency is not in explanations of social phenomena.

It is essential to the strategy of ascribing agency to groups to understand their behaviour that we can take them to generally act on true beliefs about the environment in which they operate. Otherwise, they will not effectively pursue the satisfaction of their desires, as we take them to do as rational agents. So, a group agent is viewed as a reliable truth-tracker (List and Pettit 2011, 24, 36–37). And it is understood to get this ability from the individuals constituting it. Group members, List and Pettit (2011, 36) say, function as the group’s ‘eyes and ears’. They provide the group with information about the environment just as sensory organs do to an individual.

This view becomes problematic, however, when we take seriously the fact that groups are made up of individuals pursuing their own personal ends. The problem is that individuals might have an incentive to deliberately feed their group false information, thus undermining the group’s ability to conform to a pattern of true beliefs. Let us consider a case where a group is to judge on the three propositions *p*, *p* → *q*, *q* and applies the premise-based procedure. We suppose that *p* is true and that all the group members believe this proposition. But two of the group members, B and C, have outcome-oriented preferences, as they first of all want to prevent the group from judging that *q* is the case. And since they do not care much about the group’s judgment of *p*, they both vote against *p*. The result is that the group rejects a proposition that all the members believe to be true (Moen 2019). As we see in Table 6, the majority judgments are actually consistent in this case. But by assuming the individuals lack perfect information about each other’s judgments, we can see why B and C vote strategically to ensure the group adopts their judgment of their proposition of concern.

**Table 6.** Two group members’ strategic voting leads the group to reject a proposition all group members believe to be true.

	*p*	*p* → *q*	*q*
Individual A	True	True	True
Individual B	True False	False	False
Individual C	True False	False	False
**Majority**	**True False**	**False**	**False**

The crucial point here is that we do not understand why the group rejects an obviously true proposition if we treat it as an irreducible agent. The evidence for *p* is plain for everyone to see, so why did the group fail to recognise it? The explanation is attainable only if we descend to the individual level, where we can see that because B and C wanted to avoid *q*, they had an incentive to reject *p*. From the intentional stance, then, the group is uninterpretable and appears to be malfunctioning. And as Dennett (1971, 89) notes, that suggests we should instead approach it from the physical stance, which means looking at its physical components. At this level, we can expect to find the condition that prevents normal operation. For a group, this means looking at the individual members, and we see how we can then explain the formation of a false collective belief.

Now, individual agents occasionally also form false beliefs. So, a failure to always form true beliefs cannot by itself disqualify a group for the agency. From the intentional stance, from which we ascribe agency, we should not expect to find only true beliefs. It must, however, be understandable why an agent has formed a false belief. We might, for example, find that the relevant information was difficult or costly to access (Dennett 1987, 96–97). But while this can explain why an individual forms a false belief, it will be of no use for explaining why a group forms a false belief due to its members’ strategic behaviour. The members possess the information, they just do not want to share it in their group. It is therefore problematic to assume, as List and Pettit (2011, 36) do, that individuals provide their group with reliable information, thus functioning as its ‘eyes and ears’, as they say. Only by not treating the group as an agent but instead reducing it to individual agents acting on their own motivations, can we see that these individuals need not reliably share the information they possess.

A view of individuals as strategically behaving agents thus makes us aware of a limitation to modelling groups as agents that we do not encounter when we model individuals as agents. The need for individual-level explanations, therefore, does not just undermine group-agent realism, it also reveals limitations to the use of group agency as a theoretical fiction.

## 7. Conclusion

I have considered List and Pettit’s account of group agency and whether it supports a view of group agency as a reality, not a mere fiction. I have applied a principle of parsimony that group-agent realists must satisfy to convincingly reject fictionalism. To qualify for real agency, a group’s behaviour must be shown to be explainable only by ascribing agential states to it, and not just to the individuals constituting it. Unlike List and Pettit, I have found this principle to disqualify groups as real agents. We might find it convenient to speak of groups as agents for various reasons, but such use will refer to a fiction, not a reality.

List and Pettit develop a particularly interesting defence of group-agent realism by trying to demonstrate the reality of group agency while respecting the principle of parsimony. Their account depends crucially on an argument for group autonomy. If groups control their own attitude formation, we cannot fully explain their behaviour at the individual level. This view is based on undeniable inconsistency problems of judgment aggregation. I have shown, however, that it does not follow, as List and Pettit take it to do, that collective-level procedures correcting such inconsistencies function as group minds controlling the group’s decision making. They are instead parts of the group members’ social environment affecting these individuals’ behaviour. Individuals can, as we have seen, strategically set the agenda or misrepresent their judgments to achieve their preferred outcome. We should therefore understand the social outcome to reflect individuals’ preferences, and not as the output of an autonomous group mind.

This makes group agency redundant in social explanations, and ontological parsimony does not support its inclusion in the domain of reality. We can still find it useful as a fiction, but with important limitations that become apparent from an individualist perspective.

## Footnotes

1 Hindriks (2008) takes Pettit’s account of group agency to be the most restrictive in the literature and contrasts it to accounts ascribing agency to groups more permissively.

2 Individuals’ sincere beliefs and desires might also change during group deliberation (Miller 1992). Group-agent realism, however, is based on the type of case where deliberation produces a set of attitudes that do not reflect the group members’ attitudes.

3 List and Pettit (2002) show that no non-dictatorial procedure satisfying basic conditions for fair representation of individuals’ attitudes can ensure complete and consistent collective attitudes. These conditions are universal domain (each individual can submit any complete and consistent set of judgments towards the propositions on the agenda), anonymity (all individuals’ judgments are given equal weight), and systematicity (the collective judgment of each proposition depends only on the individuals’ judgments of that proposition and not on judgments of any other proposition or on the way propositions are ordered). Any procedure satisfying these conditions while ensuring complete and consistent collective judgments (collective rationality) is a dictatorship of one individual.

4 More precisely, they show that with an agenda of inter-connected propositions, an aggregation function satisfying universal domain, collective rationality, responsiveness, and non-manipulability is a dictatorship of one individual. Responsiveness here requires that for any admissible profile, if one or more voters change their votes in some direction, while other votes remain unchanged, the collective decision cannot change in the opposite direction.

5 List and Pettit (2011, ch. 5) recognise the possibility of strategic voting, but they do not consider it a problem for their group-agent realism (see also Dietrich and List 2007b; List 2004, 505–510).

6 An implication here is that strategic voting can weaken the power of an agenda setter. We can therefore understand why it has been called the ‘flip side’ of agenda control (Riker 1986, 149).

7 List (2021, 1220) takes the more demanding view that successful ascription of agency to groups is merely a good indication that the groups are agents. For an explicitly interpretational account of group agency, see Tollefsen (2002a; 2002b; 2015, Ch. 5).

8 List and Pettit use this analogy repeatedly. See, for example, List (2014, 1616), List and Pettit (2011, 78, 161), and Pettit (2009, 86; 2014, 1654–1655).
